# Feasibility Study of a Modified XELOX Adjuvant Chemotherapy for High-Recurrence Risk Patients With Operated Stage III Colon Cancer

**DOI:** 10.3389/fphar.2020.583091

**Published:** 2020-09-18

**Authors:** Jianhong Peng, Weihao Li, Wenhua Fan, Wenhao Zhou, Ying Zhu, Xueying Li, Zhizhong Pan, Xiaoping Lin, Junzhong Lin

**Affiliations:** ^1^ Department of Colorectal Surgery, Sun Yat-sen University Cancer Center, State Key Laboratory of Oncology in South China, Collaborative Innovation Center for Cancer Medicine, Guangzhou, China; ^2^ Department of Nuclear Medicine, Sun Yat-sen University Cancer Center, State Key Laboratory of Oncology in South China, Collaborative Innovation Center for Cancer Medicine, Guangzhou, China

**Keywords:** colon cancer, adjuvant chemotherapy, XELOX (CapeOx), high risk, feasibility

## Abstract

**Background:**

Our previous study reported the favorable efficacy and good tolerance associated with a modified XELOX adjuvant chemotherapy with eight cycles of capecitabine and six cycles of oxaliplatin for operated stage III colon cancer. The current study aimed to confirm the feasibility of modified XELOX chemotherapy for treating specific high-risk (T4, N2, or both) stage III colon cancer.

**Methods:**

We selected 142 consecutive patients with high-risk stage III colon cancer who received colon tumor resection followed by modified XELOX or standard full-cycle XELOX chemotherapy from November 2007 to June 2016 at Sun Yat-sen University Cancer Center. Disease-free survival (DFS), overall survival (OS), and adverse events of patients treated with the two chemotherapy regimens were compared.

**Results:**

Seventy-four (52.1%) patients received standard XELOX chemotherapy, and 68 (47.8%) received modified XELOX chemotherapy. Neurotoxicity was the most common adverse event in 99 (69.7%) patients. Grade 2-3 neurotoxicity, grade 2–4 thrombocytopenia and grade 3–4 leucopenia were the major severe adverse events related to the decision to treat patients with modified XELOX chemotherapy. After a median follow-up of 69 months, the modified XELOX group presented a comparable 5-year DFS rate (79.0 vs. 80.3%, P = 0.891) and 5-year OS rate (93.8 vs. 87.8%, P = 0.446) as those in the standard XELOX group. Univariate survival analysis indicated that poor tumor differentiation (HR: 2.381, 95% CI: 1.141–4.968, P = 0.021) was the only significant risk factor for DFS, but no significant prognostic factor was identified for OS.

**Conclusions:**

The modified XELOX adjuvant chemotherapy presented a comparable oncologic efficacy as standard XELOX chemotherapy for high-risk stage III colon cancer. The modified XELOX adjuvant chemotherapy could be an alternative treatment for patients suffering severe adverse events, especially severe neurotoxicity.

## Introduction

To date, the combination of curative surgery and oxaliplatin-based chemotherapy is recommended as the classical treatment strategy for stage III colon cancer (Andre et al., 2009; Schmoll et al., 2014). A 6-month duration of adjuvant chemotherapy was previously recommended for all stage III colon cancer patients (Labianca et al., 2013; Benson et al., 2014). However, the International Duration Evaluation of Adjuvant Therapy (IDEA) trial introduced individualized durations of adjuvant chemotherapy according to the risk stratification of stage III colon cancer after curative chemotherapy (Grothey et al., 2018). The final results of the IDEA study suggested that a 3-month XELOX adjuvant chemotherapy regimen was sufficient for low-risk patients (T1-3 N1 disease), while 6-month XELOX adjuvant chemotherapy was still recommended for high-risk patients (T4, N2, or both). In fact, chemotherapy-related toxicity, especially neurotoxicity, causes 30–50% of patients to be unable to finish the full planned duration of oxaliplatin-containing adjuvant chemotherapy (Alberts et al., 2012; Lonardi et al., 2016). Therefore, a shorter duration of oxaliplatin-containing adjuvant therapy for stage III patients is warranted, especially for high-risk patients.

Recently, we reported that a modified XELOX (mXELOX) adjuvant chemotherapy with 8 cycles of capecitabine and 6 cycles of oxaliplatin presented a comparable survival outcome and lower incidence of neurotoxicity than a standard full cycle of XELOX chemotherapy for patients with stage III operated colon cancer (Peng et al., 2019). Although the previous study provided the first evidence supporting the administration of the mXELOX adjuvant chemotherapy regimen for stage III colon cancer patients, it did not assess the long-term oncologic efficacy of modified XELOX chemotherapy in those patients by performing a comparison with the standard full cycle of XELOX chemotherapy. Nevertheless, based on the results of the study, we supposed that mXELOX adjuvant chemotherapy could reduce or prevent the aggravation of neurotoxicity in patients with high-risk stage III colon cancer without impairing their oncologic outcomes.

To further demonstrate the feasibility of modified XELOX chemotherapy for treating high-risk stage III colon cancer, the present study aimed to evaluate the oncologic efficacy and safety of modified XELOX chemotherapy for these patients.

## Materials and Methods

### Patients

This retrospective study reviewed 448 consecutive patients with colon cancer who underwent primary tumor resection between November 2007 and June 2016 at Sun Yat-sen University Cancer Center, China.The 448 patients met the following criteria: (1) pathologically diagnosed with colon adenocarcinoma, (2) underwent colon tumor curative resection, (3) received adjuvant chemotherapy with the XELOX regimen (oxaliplatin 130 mg/m^2^ administered intravenously on day 1 and capecitabine 1,000 mg/m^2^ administered orally twice daily on days 1–14 for a 3-week cycle), (4) received no preoperative anticancer treatment, (5) was categorized as American Society of Anesthesiologists class I–II, (6) attended postoperative follow-up at least 3 months after delivery of the first cycle of chemotherapy, and (7) had a complete record of chemotherapy side effects. Subsequently, we further selected the patients according to the following criteria: (1) high-recurrence-risk patients with stage pT4Nany or pTanyN2 and (2) patients who underwent adjuvant chemotherapy with the full-cycle standard XELOX or the modified XELOX regimen. The clinical information, including demographics, tumor characteristics, treatment details, and follow-up data, was carefully collected from the electronic medical record system. The current study was conducted based on the ethical standards of the World Medical Association Declaration of Helsinki. The study was approved by the Institutional Research Ethics Committee of Sun Yat-sen University Cancer Center (approval number: GZR2017-004). Informed consent was waived by independent ethics committees of Sun Yat-sen University Cancer Center because of the retrospective nature of the study. All patient data were documented confidentially.

### Treatments

All patients underwent curative resection of the colon tumor by the performance of standard complete mesocolic excision and regional lymphadenectomy. The initial adjuvant chemotherapy was performed 3–8 weeks after colon tumor resection for all patients. According to the cycles of oxaliplatin given, patients with adjuvant chemotherapy was grouped into the standard and modified XELOX regimen group. Modified XELOX was defined as six cycles of the XELOX regimen plus two subsequent cycles of capecitabine alone, which consisted of eight cycles of capecitabine and six cycles of oxaliplatin, while the standard XELOX regimen consisted of eight cycles of capecitabine and eight cycles of oxaliplatin. The administration of the two XELOX regimens of adjuvant chemotherapy depended on the toxicity of the chemotherapy, the patient’s tolerance, and preferences for the last two cycles of chemotherapy. A complete laboratory assessment was performed before each chemotherapy cycle.

### Definitions

All cases were pathologically staged according to the 8^th^ edition of the American Joint Committee on Cancer (AJCC) staging system. Right-sided colon cancer was defined as a tumor located in the cecum, ascending colon, hepatic flexure, or transverse colon. Left-sided colon cancer was recognized as a tumor in the splenic flexure, descending colon, and sigmoid colon (Peng et al., 2018). The lymph node ratio (LNR) was defined as the number of positive lymph nodes divided by the total number of retrieved lymph nodes. Lymphovascular invasion (LVI) was diagnosed based on the presence of tumor cells within the small endothelium-lined lymphatic or vascular channels (Harris et al., 2008). Perineural invasion (PNI) was diagnosed based on tumor invasion in, around, and through nerves and nerve sheaths (Batsakis, 1985). The intensity of the adverse events during adjuvant chemotherapy was graded based on the National Cancer Institute Common Terminology Criteria for Adverse Events (NCI CTCAE), version 4.0.

### Postoperative Follow-Up

Follow-up was conducted through clinical visits every 3 months for the first 2 years and then semiannually for the subsequent 3 years after surgery. The clinical visit items included abdominal examinations, detection of serum carcinoembryonic antigen (CEA) and carbohydrate antigen 19-9 (CA19-9), chest/abdominal/pelvic CT, and colonoscopy. Disease-free survival (DFS) was the interval from the date of tumor resection to the date of disease recurrence, death or the last follow-up. Overall survival (OS) was the interval from the date of tumor resection to the date of death from any cause or the last follow-up. The final follow-up visit was conducted in July 2019.

### Statistical Analysis

Statistical analyses were conducted using SPSS 20.0 software (IBM, Chicago, IL, USA) and GraphPad Prism 7 software (GraphPad Software, Inc., San Diego, CA, USA). Continuous variables are presented as the median (range), while categorical variables are presented as percentages, which were compared by using the chi-square (χ^2^) test. The Kaplan–Meier curve was applied to calculate the survival rates, and the differences in survival between the groups were subsequently compared by using the log-rank test. The hazard ratios (HRs) and 95% confidence intervals (CIs) were finally generated by univariate Cox proportional hazards analysis. The statistical tests performed above were two sided, and a P value less than 0.05 was considered significant.

## Results

### Patient Characteristics

**Figure 1:**
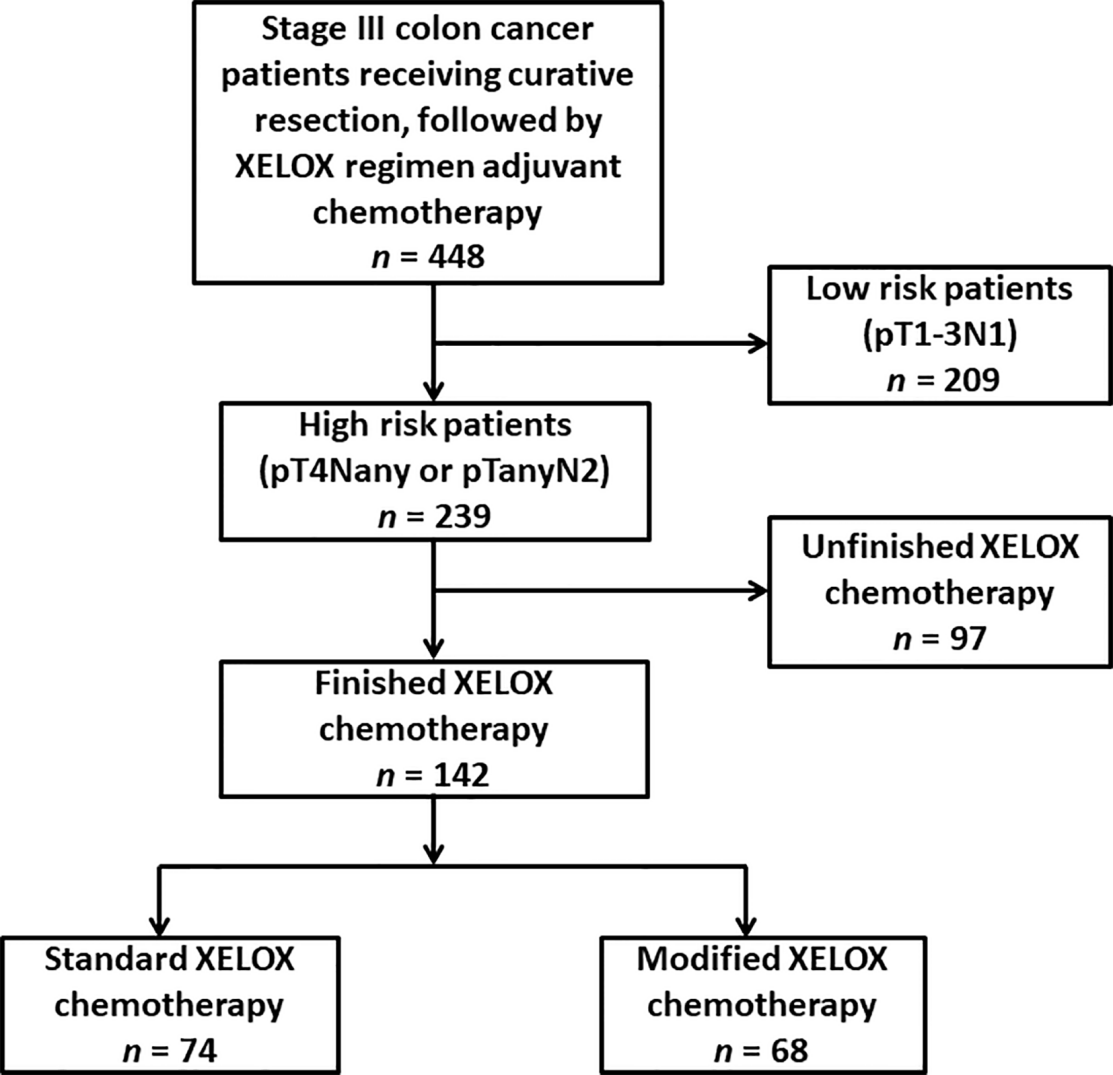
Flowchart representing the selection of eligible patients for the present study.

The participant selection flowchart is presented in **Figure 1**. Among the 448 patients, 306 patients were excluded for the following reasons: low-recurrence risk patients (n = 209) and unfinished adjuvant chemotherapy with fewer than eight cycles (n = 97). Overall, 142 eligible patients were identified for analysis in the study, with 74 patients in the standard XELOX group and 68 patients in the modified XELOX group. The detailed information of the 142 patients is shown in **Table 1**. The median age of the total sample of patients was 55years (range, 22–85 years), with 56.3% male patients. The median tumor size was 4.1 cm (range, 1.0–12.0 cm). The median number of retrieved lymph nodes was 15 (range, 2–63), the median number of metastatic lymph nodes was 3 (range, 1–17), and the median LNR was 0.25 (range, 0.09–1.00). According to tumor location, the total sample of patients had 3 (2.1%) cases of cecal cancer, 20 (14.1%) cases of ascending colon cancer, 11 (7.7%) cases of hepatic ﬂexure colon cancer, 10 (7.0%) cases of transverse colon cancer, 4 (2.8%) cases of splenic ﬂexure colon cancer, 18 (12.7%) cases of descending colon cancer, and 76 (53.5%) cases of sigmoid colon cancer. In total, three (2.1%) patients experienced postoperative complications such as intestinal obstruction.

**Table 1:** Clinical characteristics of the patients in the standard and modified XELOX groups.

Variable	Overall cases (%)	Standard XELOX (n, %)	Modified XELOX (n, %)	P value
Total	142	74 (52.1)	68 (47.9)	
Age (years)				0.487
≤60	100 (70.4)	54 (73.0)	46 (67.6)	
>60	42 (29.6)	20 (27.0)	22 (32.4)	
Sex				0.657
Male	80 (56.3)	43 (58.1)	37 (54.4)	
Female	62 (43.7)	31 (41.9)	31 (45.6)	
Tumor location				**0.031**
Right-sided colon	44 (31.0)	17 (23.0)	27 (39.7)	
Left-sided colon	98 (69.0)	57 (77.0)	41 (60.3)	
Baseline hemoglobin (g/L)				0.825
<90	24 (16.9)	13 (17.6)	11 (16.2)	
≥90	118 (83.1)	61 (82.4)	57 (83.8)	
Tumor size (cm)				**0.019**
≤4	71 (50.0)	30 (40.5)	41 (60.3)	
>4	71 (50.0)	44 (59.5)	27 (39.7)	
Differentiation				0.437
Well/moderate	100 (70.4)	50 (67.6)	50 (73.5)	
Poor/undifferentiated	42 (29.6)	24 (32.4)	18 (26.5)	
T stage				**0.002**
T1–T3	28 (19.7)	22 (29.7)	6 (8.8)	
T4	114 (80.3)	52 (70.3)	62 (91.2)	
Number of retrieved lymph nodes				0.207
<12	35 (24.6)	15 (20.3)	20 (29.4)	
≥12	107 (75.4)	59 (79.7)	48 (70.6)	
LNR				0.693
<0.25	79 (24.6)	40 (54.1)	39 (57.4)	
≥0.25	63 (75.4)	34 (45.9)	29 (42.6)	
N stage				0.492
N1	73 (55.6)	36 (48.6)	37 (54.4)	
N2	69 (44.4)	38 (51.4)	31 (45.6)	
LVI^a^				0.821
Positive	50 (40.0)	26 (38.2)	24 (42.1)	
Negative	75 (60.0)	42 (61.8)	33 (57.9)	
PNI^b^				0.885
Positive	38 (30.6)	21 (31.3)	17 (29.8)	
Negative	86 (69.4)	46 (68.7)	40 (70.2)	
Mismatch repair protein status^c^				0.970
pMMR	57 (91.9)	46 (92.0)	11 (91.7)	
dMMR	5 (8.1)	4 (8.0)	1 (8.3)	
Preoperative serum CEA (ng/ml)				**0.008**
≤5	84 (59.2)	36 (48.6)	48 (70.6)	
>5	58 (40.8)	38 (51.4)	20 (29.4)	
Postoperative metastasis				0.643
Yes	29 (20.4)	14 (18.9)	15 (22.1)	
No	113 (79.6)	60 (81.1)	53 (77.9)	

LNR, lymph node ratio; LVI, lymphovascular invasion; PNI, perineural invasion; pMMR, proficient mismatch repair protein; dMMR, deficient mismatch repair protein; CEA, carcinoembryonic antigen. ^a^Data from 125 patients were available; ^b^Data from 124 patients were available. ^c^Data from 62 patients were available. It will be bold when the p-value result is less than 0.05.

### Differences in Clinicopathological Parameters Between the Two XELOX Regimens

As shown in **Table 1**, the modified XELOX group presented a significantly higher prevalence of T4 (91.2 vs. 70.3%; P = 0.002) and right-sided colon tumors (39.7 vs. 23.0%; P = 0.031) than those in the standard XELOX group. However, the standard XELOX group showed a significantly higher prevalence of larger tumors (59.5% vs. 39.7%; P = 0.019) and elevated preoperative CEA levels (51.4 vs. 29.4%; P = 0.008) than those in the modified XELOX group. The differences in other clinicopathological parameters between the groups did not show statistical significance.

### Adverse Events of Adjuvant Chemotherapy

**Figure 2:**
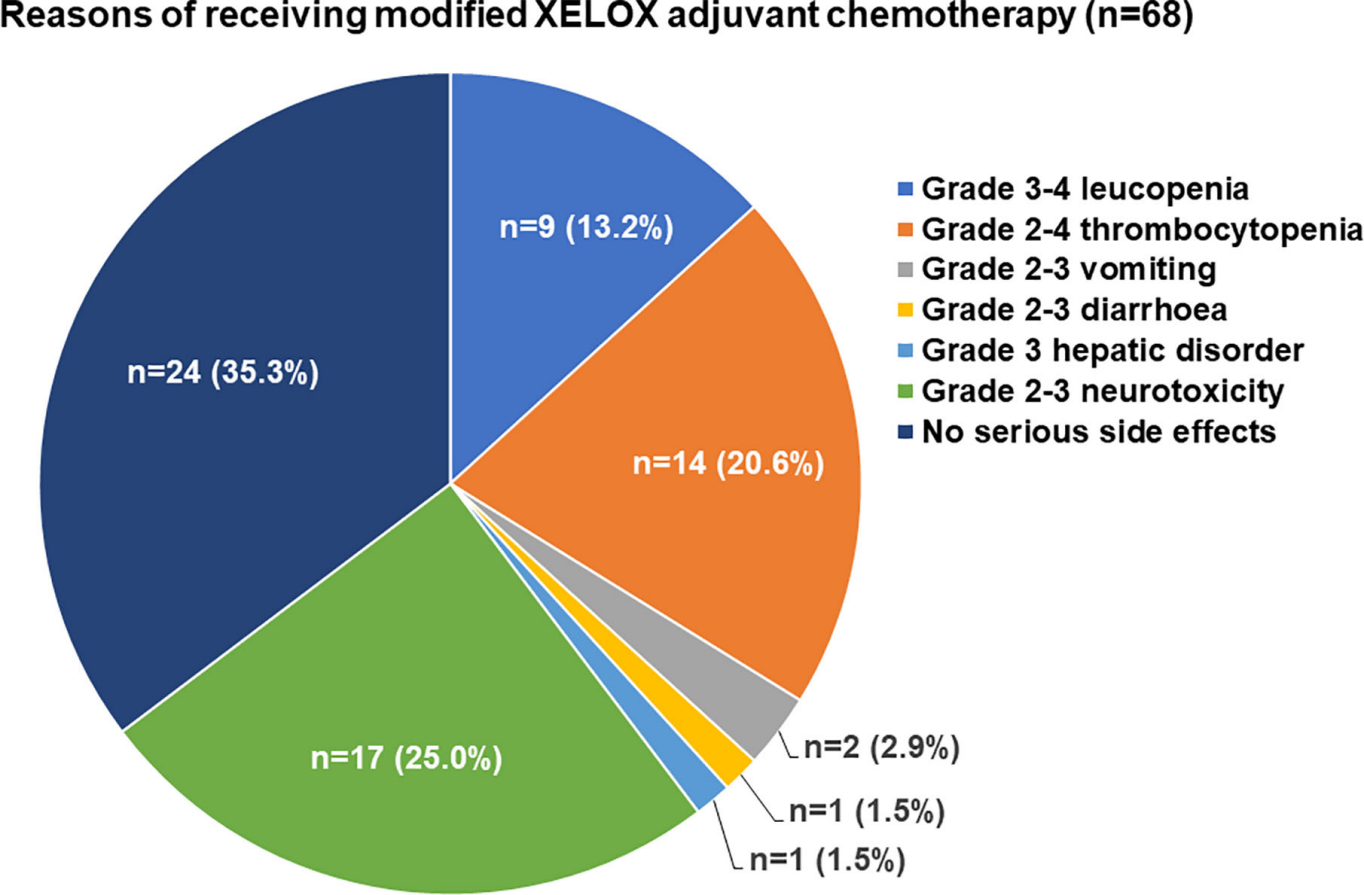
Reasons of receiving modified XELOX adjuvant chemotherapy.

The major adverse events related to adjuvant chemotherapy with the two regimens are presented in **Table 2**. There were no deaths due to grade 3/4 adverse events. Neurotoxicity was found in 99 (69.7%) patients and was the most common adverse event among all patients. Compared with the standard XELOX group, the modified XELOX group showed a lower incidence of grade 1 hepatic disorder (14.7 vs. 28.4%, P = 0.049). There were no significant differences in the total occurrence rates of leukopenia, thrombocytopenia, hepatic disorder, nausea and vomiting, diarrhea, neurotoxicity, or hand-foot syndrome between the two groups. The reasons for the decision to treat patients with modified XELOX chemotherapy are presented in **Figure 2**. The decision to treat patients with modified XELOX chemotherapy was mostly due to severe adverse events in 64.7% (44/68) of patients. Grade 2–3 neurotoxicity (25.0%, 17/68), grade 2–4 thrombocytopenia (20.6%, 14/68), and grade 3–4 leukopenia (13.2%, 9/68) were the major severe adverse events related to the decision to treat patients with modified XELOX chemotherapy. No aggravation of neurotoxicity was observed in the last two chemotherapy cycles in the modified XELOX group.

**Table 2:** Comparison of chemotherapy-related toxicities between the standard and modified XELOX groups.

Toxicity	Total patients (n = 142, %)	Standard XELOX (n = 74, %)	Modified XELOX (n = 68, %)	P value
Leucopenia				
Total	89 (62.7)	47 (63.5)	42 (61.8)	0.830
Grade 1-2	77 (54.2)	41 (55.4)	36 (52.9)	0.768
Grade 3-4	12 (8.5)	6 (8.1)	6 (8.8)	0.878
Thrombocytopenia				
Total	67 (47.2)	35 (47.3)	32 (47.1)	0.977
Grade 1-2	52 (36.6)	29 (39.2)	23(33.8)	0.507
Grade 3-4	15 (10.6)	6 (8.1)	9 (13.2)	0.321
Hepatic disorder				
Total	45 (31.7)	28 (37.8)	17 (25.0)	0.100
Grade 1	31 (21.8)	21 (28.4)	10 (14.7)	**0.049**
Grade 2-3	14 (9.9)	7 (9.5)	7 (10.3)	0.868
Nausea/vomiting				
Total	38 (26.8)	18 (24.3)	20 (29.4)	0.494
Grade 1	22 (15.5)	11(14.9)	11 (16.2)	0.829
Grade 2-3	16 (11.3)	7 (9.5)	9 (13.2)	0.477
Diarrhea				
Total	22 (15.5)	12 (16.2)	10 (14.7)	0.804
Grade 1	12 (8.5)	6 (8.1)	6 (8.8)	0.878
Grade 2-3	10 (7.0)	6 (8.1)	4 (5.9)	0.605
Neurotoxicity				
Total	99 (69.7)	47(63.5)	52 (76.5)	0.093
Grade 1	64 (45.1)	32 (43.2)	32 (47.1)	0.648
Grade 2-3	35 (24.6)	15 (20.3)	20 (29.4)	0.207
Hand-foot syndrome				
Total	78 (54.9)	37 (50.0)	41 (60.3)	0.218
Grade 1	63 (44.3)	30 (40.5)	33 (48.5)	0.338
Grade 2-3	15 (10.6)	7 (9.5)	8 (11.8)	0.655

The listed grades of peripheral sensory neurotoxicity represent the maximal levels at any time. It will be bold when the p-value result is less than 0.05.

### Survival Analysis

**Figure 3:**
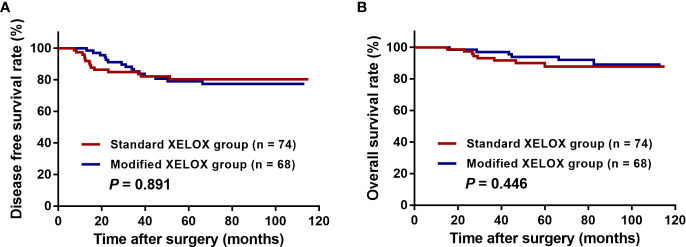
Kaplan-Meier curves of patients with high-risk stage III colon cancer grouped by standard XELOX and modified XELOX group. **(A)** Comparison of disease-free survival (DFS) between the standard XELOX and modified XELOX group. **(B)** Comparison of overall survival (OS) between the standard XELOX and modified XELOX group.

After a median postoperative follow-up duration of 69 months (range, 7–115 months), 29 (20.4%) patients developed disease recurrence, and 14 (9.9%) patients ultimately experienced cancer-related mortality. Among these patients, 44.8% (13/29) had liver metastases, 31.0% (9/29) had lung metastases, 27.6% (8/29) had abdominal pelvic metastases, and 17.2% (5/29) had metastases to other organs. The 5-year DFS and OS rates were 79.7 and 90.9%, respectively, among all enrolled patients in this study. The 5-year DFS rate was 80.3% for patients in the standard XELOX group and 79.0% for patients in the modified XELOX group; these values were comparable (P = 0.891) (**Figure 3A**). Similarly, the 5-year OS rate was 87.8% for the standard XELOX group and 93.8% for the modified XELOX group; these values were not significantly different (P = 0.446) (**Figure 3B**). In univariate analysis, poor tumor differentiation (HR: 2.381, 95% CI: 1.141–4.968, P = 0.021) was the only significant risk factor for DFS, while there was no significant prognostic factor for OS (**Table 3**).

**Table 3:** Univariate analyses of prognostic factors for disease-free survival and overall survival in all patients.

Variable	DFS		OS	
	Univariate		Univariate	
	HR (95%CI)	P value	HR (95%CI)	P value
Age, years (>60 vs. ≤60)	2.081 (1.001–24.329)	0.050	0.953 (0.299–3.039)	0.935
Sex (Male vs. Female)	1.952 (0.888–4.288)	0.096	1.546 (0.517–4.621)	0.435
Baseline hemoglobin, g/l (<90 vs. ≥90)	0.493 (0.149–1.630)	0.246	0.035 (0.000–10.900)	0.253
Tumor location (left-sided colon vs. right-sided colon)	1.385 (0.592–3.242)	0.453	0.575 (0.199–1.660)	0.306
Tumor size, cm (>4 vs. ≤4)	0.589 (0.278–1.248)	0.167	0.570 (0.191–1.702)	0.314
Differentiation (Poor vs. Well-moderate)	2.381 (1.141–4.968)	**0.021**	1.760 (0.585–5.298)	0.315
T stage (T4 vs. T1-3)	1.525 (0.530–4.388)	0.434	2.689 (0.349–20.722)	0.343
Number of retrieved lymph nodes (<12 vs. ≥12)	1.649 (0.767–3.547)	0.201	0.286 (0.792–6.599)	0.126
LNR (≥0.25 vs. <0.25)	2.086 (0.976–4.458)	0.058	1.816 (0.627–5.261)	0.272
N stage (N2 vs. N1)	0.888 (0.427–1.847)	0.427	0.857 (0.296–2.476)	0.775
LVI (positive vs. negative)	1.339 (0.600–2.989)	0.476	1.137 (0.361–3.582)	0.827
PNI (positive vs. negative)	1.399 (0.612–3.198)	0.426	1.197 (0.360–3.981)	0.769
Preoperative CEA, ng/ml (>5 vs. ≤5)	1.393 (0.672–2.886)	0.373	1.125 (0.390–3.244)	0.827
Adjuvant chemotherapy (modified XELOX vs. standard XELOX)	1.052 (0.507–2.184)	0.891	0.660 (0.226–1.930)	0.448

LNR, lymph node ratio; LVI, lymphovascular invasion; PNI, perineural invasion; CEA, carcinoembryonic antigen. It will be bold when the p-value result is less than 0.05.

## Discussion

Based on the results of our previous study, the current study further evaluated the long-term efficacy of modified XELOX chemotherapy for the treatment of high-risk stage III colon cancer. In line with our hypothesis, the results indicated that modified XELOX chemotherapy had oncologic efficacy for high-risk patients comparable to that of standard XELOX chemotherapy. Furthermore, modified XELOX showed acceptable safety without aggravating neurotoxicity. The study enhanced the evidence supporting the use of modified XELOX adjuvant chemotherapy for stage III operated colon cancer patients, including the high-risk subgroup.

To our knowledge, the unfavorable prognostic impact of advanced T and N stages for stage III cancer patients has been well identified. Although curative surgery provided temporary evidence of absence of disease for those patients, residual micrometastases probably exist due to the advanced disease stage, which increases the likelihood of disease recurrence postoperatively (Viehl et al., 2017). Based on this theory, these patients warrant aggressive postoperative chemotherapy to maximize the survival benefit from curative treatment. Evidence from the France IDEA trial supported that high-risk patients achieve greater survival benefit from the 6-month full cycle of adjuvant chemotherapy instead of the 3-month duration of adjuvant chemotherapy (Andre et al., 2018). Previous studies have also shown that failure to complete adjuvant chemotherapy impairs long-term survival in stage III colon cancer patients (Morris et al., 2007), which demonstrates the necessary of finishing adjuvant 5-FU–based chemotherapy for colon cancer. In our study, the total sample of patients achieved favorable long-term survival, with 79.7% 5-year DFS and 90.9% 5-year OS in both the standard XELOX group and modified XELOX group finishing the 6-month 5-FU–based adjuvant chemotherapy. Therefore, we considered a 6-month duration of 5-FU–based adjuvant chemotherapy to be necessary for disease control in high-risk patients.

On the other hand, the choice of regimen should also be balanced against the additional toxicity associated with longer therapy. Cumulative evidence has reported that approximately 70% of patients develop neurotoxicity during treatment with oxaliplatin-containing chemotherapy (Land et al., 2007). Similarly, nearly 70% of patients suffered oxaliplatin-related neurotoxicity and 24.6% of patients experienced severe neurotoxicity in the current study. It has been proven that neurotoxicity accumulates with the increase in oxaliplatin administration, leading to poor compliance in the subsequent chemotherapy course (Pachman et al., 2015). More seriously, the neurotoxicity contributing to sensory nerve deficits might persist for years after cessation of oxaliplatin therapy (Kokotis et al., 2016). Obviously, continuous treatment with oxaliplatin increased the risk of developing new neurotoxicity or worsening previous neurotoxicity (Besora et al., 2018). The JSWOG-C2 Study demonstrated the feasibility of a sequential approach to adjuvant chemotherapy with 3 months of an oxaliplatin-based regimen followed by 3 months of capecitabine in stage III and high-risk stage II colorectal cancer patients, which was tolerated by patients and associated with a low incidence of neuropathy (Tsuruta et al., 2016). Another phase 2 Japanese study also reported that intermittent oxaliplatin treatment improved severe neuropathy in a modified FOLFOX6 plus bevacizumab regimen without reducing progression-free survival in patients with inoperable or metastatic colorectal cancer (Kato et al., 2018). In fact, we previously found that a two-cycle shorter duration of oxaliplatin treatment in the modified XELOX regimen was associated witha significantly lower incidence and severity of adverse events, especially neurotoxicity, without impairing survival (Peng et al., 2019). In the current study, 64.7% (44/68) of patients suffered severe adverse events, of which grade 2–3 neurotoxicity was the most common severe adverse event. Those patients who were initially predicted to fail to finish the full cycle of adjuvant chemotherapy successfully finished the 6-month adjuvant chemotherapy due to the administration of the modified XELOX regimen. This might be explained mostly by the lack of aggravating neurotoxicity in the last two chemotherapy cycles with capecitabine alone. Furthermore, our results revealed that modified XELOX chemotherapy did not impair 5-year survival compared with the survival associated with standard XELOX chemotherapy. Based on the results of the comparable oncologic efficacy, a two-cycle shorter duration of oxaliplatin treatment might be an alternative approach for high-risk stage III colon cancer patients with severe adverse events, especially severe neurotoxicity.

Several limitations to the current study should be acknowledged. First, this retrospective study was performed with an uncontrolled methodology and a limited number of patients in a single cohort. It can not be denied that there exists selective bias such as patients in standard XELOX group tend to have more high-risk clinical characteristics likes bigger tumor size, more T4 stage, higher CEA level and have better tolerance to chemotherapy toxicity. Although our study initially indicated the oncologic efficacy of modified XELOX chemotherapy, more work should be done in a prospective, multicenter clinical trial with a large sample size to validate these findings in the future. Second, we failed to evaluate the long-term quality of life effects of the two chemotherapy regimens. As a result, we were unable to compare the occurrence of late effects of oxaliplatin-induced peripheral neuropathy between the two chemotherapy groups. Since persistent neuropathy impaired the survivors’ physical and emotional well-being, it is an important parameter to measure the feasibility of an oxaliplatin-containing chemotherapy regimen (Tofthagen et al., 2013). Third, although the TNM stage is important for colon cancer management, it seems to be insufficient to determine the authentic high-risk patients with stage III colon cancer. Tumor molecular markers, such as CpG island methylator phenotype (CIMP) status; driver gene mutations, such as KRAS and BRAF; and tumor immune microenvironment, have been linked to different recurrence risks among stage III colon cancer patients (Auclin et al., 2017; Murcia et al., 2018). The above molecular data were unavailable in the current study. Therefore, molecular prognostic markers for risk stratification need to be explored in further works.

## Conclusion

Our study confirms that modified XELOX chemotherapy presented comparable oncologic efficacy for patients with high-risk stage III colon cancer. These findings indicated that modified XELOX chemotherapy could serve as an alternative regimen for patients suffering severe adverse events, especially severe neurotoxicity.

## Data Availability Statement

The datasets used and analyzed during the current study are available from the corresponding author on reasonable request. The authenticity of this article has been validated by uploading the key raw data onto the Research Data Deposit public platform (http://www.researchdata.org.cn), with the Approval Number as RDDA2020001633.

## Ethics Statement

The studies involving human participants were reviewed and approved by the Institutional Research Ethics Committee of Sun Yat-sen University Cancer Center (approval number: GZR2017-004). Written informed consent for participation was not required for this study in accordance with the national legislation and the institutional requirements.

## Author Contributions

All authors listed have made a substantial, direct, and intellectual contribution to the work and approved it for publication.

## Funding

The study was funded by grants from the National Natural Science Foundation of China (no. 81772595 and no. 81871991), Natural Science Foundation of Guangdong Province, China (no. 2018A030310239), and Bethune-Ethicon Excellence Surgery Fund Project (no. HZB-20181119-27).

## Conflict of Interest

The authors declare that the research was conducted in the absence of any commercial or financial relationships that could be construed as a potential conflict of interest.
